# The Effects of Oxytocin on Appetite Regulation, Food Intake and Metabolism in Humans

**DOI:** 10.3390/ijms22147737

**Published:** 2021-07-20

**Authors:** Liya Kerem, Elizabeth A. Lawson

**Affiliations:** 1 Neuroendocrine Unit, Department of Medicine, Massachusetts General Hospital and Harvard Medical School, Boston, MA 02114, USA; liya.em@gmail.com; 2 Division of Pediatric Endocrinology, Massachusetts General Hospital for Children, Boston, MA 02114, USA

**Keywords:** oxytocin, body weight, appetite regulation, energy expenditure, food intake, metabolism, fMRI

## Abstract

The hypothalamic peptide oxytocin and its receptor are involved in a range of physiological processes, including parturition, lactation, cell growth, wound healing, and social behavior. More recently, increasing evidence has established the effects of oxytocin on food intake, energy expenditure, and peripheral metabolism. In this review, we provide a comprehensive description of the central oxytocinergic system in which oxytocin acts to shape eating behavior and metabolism. Next, we discuss the peripheral beneficial effects oxytocin exerts on key metabolic organs, including suppression of visceral adipose tissue inflammation, skeletal muscle regeneration, and bone tissue mineralization. A brief summary of oxytocin actions learned from animal models is presented, showing that weight loss induced by chronic oxytocin treatment is related not only to its anorexigenic effects, but also to the resulting increase in energy expenditure and lipolysis. Following an in-depth discussion on the technical challenges related to endogenous oxytocin measurements in humans, we synthesize data related to the association between endogenous oxytocin levels, weight status, metabolic syndrome, and bone health. We then review clinical trials showing that in humans, acute oxytocin administration reduces food intake, attenuates fMRI activation of food motivation brain areas, and increases activation of self-control brain regions. Further strengthening the role of oxytocin in appetite regulation, we review conditions of hypothalamic insult and certain genetic pathologies associated with oxytocin depletion that present with hyperphagia, extreme weight gain, and poor metabolic profile. Intranasal oxytocin is currently being evaluated in human clinical trials to learn whether oxytocin-based therapeutics can be used to treat obesity and its associated sequela. At the end of this review, we address the fundamental challenges that remain in translating this line of research to clinical care.

## 1. Introduction

The neuropeptide oxytocin and its receptor are well-documented for orchestrating a broad repertoire of physiological processes across species. Oxytocin was first discovered in 1906 for its uterine-contracting properties [1] and shortly after, its involvement in lactation was described [2,3]. Since the beginning of the 20th century, numerous studies have revealed the involvement of oxytocin in a range of biological functions, including cell growth and regulation, wound healing, and anti-inflammatory actions as well as social behaviors such as mother–infant attachment and pair-bonding [4,5,6,7,8]. Along this line of research, compelling evidence has accumulated, establishing the role of oxytocin in appetite regulation, eating behavior, weight status, and metabolism in both animal models and humans [9,10,11,12,13]. The concerning increase in obesity prevalence and its associated serious metabolic derangements [14] has spurred investigational efforts to study oxytocin given its anorexigenic properties [15], beneficial metabolic and weight loss effects [12], and minimal side effects. Human studies are currently ongoing to assess the promise of oxytocin as a therapeutic agent and to evaluate the potential involvement of disrupted oxytocin actions in the pathophysiology of obesity and metabolic syndrome.

In this review, we describe the distribution of oxytocin and its receptor in the central nervous system (CNS) as a framework to mechanistically explain the involvement of oxytocin in eating behavior and energy balance. We then continue to discuss the peripheral effects oxytocin exerts through the oxytocin receptor (OXTR), including those related to the musculoskeletal tissue, to adipose tissue metabolism, and in regard to glucose homeostasis. Next, we synthesize key findings derived from animal studies that established the therapeutic potential of oxytocin and laid the groundwork for translational research in humans. Prior to presenting findings from observational studies in humans aimed at characterizing the association between circulating endogenous oxytocin and metabolic health, we delineate the different strategies available to measure oxytocin and the resulting difficulties in compiling the different studies to form a clear understanding of this association. Later in the review, we focus on interventional human studies assessing the effects of oxytocin on food intake, neurobiological processing of food stimuli, and metabolism. Genetic conditions and brain pathologies associated with oxytocin depletion are discussed to emphasize the role of oxytocin in eating behavior and energy balance. Finally, we discuss the knowledge gaps that remain to be bridged prior to utilizing oxytocin as a therapeutic agent in obesity and metabolic syndrome.

## 2. The Central Oxytocinergic System—A Framework to Understand the Role of Oxytocin in the Coordination of Energy Balance

Oxytocin is a nine-amino acid neuropeptide hormone that is highly conserved across phyla [6]. It is synthesized in conjunction with a carrier protein (neurophysin 1) and cleaved from oxytocin precursors or extended forms of oxytocin, which consist of 10–12 amino acids [16]. Oxytocin is predominantly produced in the hypothalamus, a brain region that has a critical role in neuroendocrine homeostatic regulation of energy consumption and expenditure [17]. Specific nuclei of the hypothalamus (e.g., arcuate nucleus (ARC), paraventricular nucleus (PVN), ventromedial hypothalamic nucleus) integrate peripheral (e.g., adipose tissue, gastrointestinal (GI) tract), endocrine, nutritional, and metabolic signals reflecting energy consumption and reserves with higher order neural input to control satiety, hunger, and energy balance [18]. Oxytocin is synthesized in PVN parvocellular neurons and by magnocellular neurosecretory cells located in both the PVN and supraoptic (SON) nuclei of the hypothalamus [19,20].

**Figure 1:**
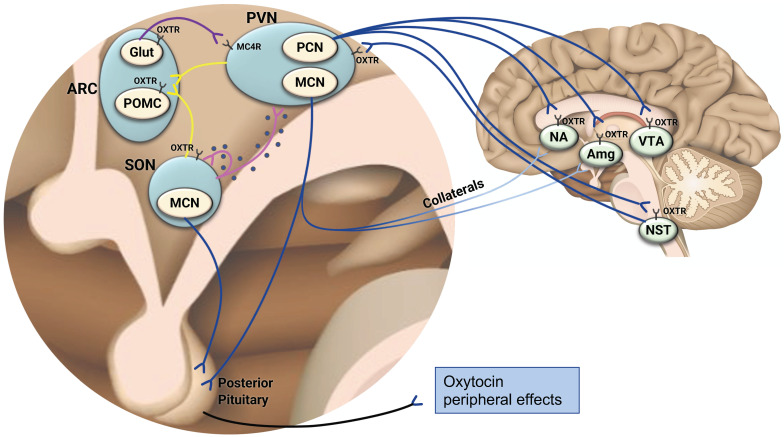
The participation of oxytocin in appetite regulation and metabolism. Oxytocin is produced in parvocellular neurons located in the paraventricular nucleus (PVN) and in magnocellular neurons located in the PVN and supraoptic nucleus (SON). Shown in the figure are some of the oxytocinergic pathways affecting appetite and metabolism. Glutamate releasing ARC neurons that express the OXTR and project to melanocortin 4 receptor (MC4R) expressing neurons in the PVN (purple lines) induce rapid satiation when chemo- or optogenetically stimulated. Oxytocinergic neurons originating from the PVN and SON and reaching POMC OXTR expressing ARC neurons (yellow lines) also induce satiation. Somato-dendritic secretion of oxytocin from magnocellular cells (pink arrows) results in autocrine and paracrine local effects. Axonal oxytocinergic projections from the PVN (blue lines) reach multiple brain regions involved in shaping eating behavior (shown in the figure is the ventral tegmental area (VTA), nucleus accumbens (NA), amygdala (Amg), and the nucleus of solitary tract (NST)). Oxytocin synthesized in magnocellular cells is carried by axonal transport to the posterior pituitary (blue lines), where it is stored until release into the systemic circulation. Peripheral circulating oxytocin affects multiple organs to exert metabolic effects inducing lipolysis, increased fatty acid beta oxidation, brown adipose tissue thermogenesis, bone mineralization, and skeletal muscle regeneration.

Within the brain, oxytocin signaling takes place by two categorical mechanisms (see Figure 1). First, somato-dendritic secretion of oxytocin from magnocellular cells results in autocrine and paracrine local effects [21]. Large dense-cored vesicles (LDCV) containing oxytocin can be found distributed throughout oxytocin neurons and are released by exocytosis, which is regulated by intracellular Ca^2+^ [19,22]. Additionally, oxytocin itself can elicit dendritic peptide release by activating oxytocin receptors located on oxytocin neurons’ dendrites or soma, thus eliciting calcium release from thapsigargin-sensitive intracellular stores, which triggers exocytosis of LDCVs containing oxytocin [23]. This mechanism of oxytocin release is needed to generate synchronized electrical activity of the magnocellular cells, which results in pulsatile secretion of oxytocin into the systemic circulation, e.g., to induce uterine contractions during labor and intramammary pressure during suckling [22,24]. In addition to its autocrine effects, oxytocin released by somato-dendritic secretion may act on brain areas located in proximity to the PVN and SON, such as the ventromedial hypothalamus [21]. Secondly, oxytocin exerts distal effects within the brain via axonal projections originating from mostly parvocellular PVN neurons and reaching multiple brain areas that are involved in the control of eating behavior [11,25] (Figure 1). Very recently, a subset of magnocellular neurons that also sends axonal projections within the brain has been discovered and is discussed subsequently [26]. Among the brain areas which the oxytocinergic neurons reach is the adjacent ARC [27], a major hub of appetite regulation which houses first-order anorexigenic neurons that express pro-opiomelanocortin (POMC) and cocaine- and amphetamine-regulated transcript to induce satiety, along with orexigenic neurons that signal hunger by releasing Neuropeptide Y (NPY) and Agouti Related Peptide (AgRP) [17,28]. These neurons sense alterations in metabolic status by receiving adiposity-related (e.g., leptin), nutrient (e.g., glucose), and gut-derived endocrine (e.g., ghrelin, Pancreatic Tyrosine; (PYY)) signals [29]. In addition to the oxytocinergic homeostatic neurocircuitry within the hypothalamus, oxytocin neurons project to brain regions that function as key mediators of reward seeking and consumption behavior (i.e., ventral tegmental area (VTA) [30] and the nucleus accumbens (NAc)) [31] that have been implicated in the pathogenesis of obesity and drug addiction [32,33]. Furthermore, hypothalamic oxytocinergic neurons reach the central amygdala [34,35], which modulates food consumption by affecting the reward saliency of food through positive-valence mechanisms [36]. PVN oxytocinergic neurons also project to the nucleus of the solitary tract (NST) [37] in which GI vagal afferent transmissions are integrated with endocrine peripheral signals and nutrients (e.g., glucose) to process and govern energy balance [38]. The characteristics of the neurotransmission that occurs between the hypothalamic oxytocinergic neurons and these different brain areas are complex and depend on the target region to which they project [6]. For instance, optogenetic stimulation of PVN oxytocinergic terminals reaching dopaminergic (DA) midbrain areas directly increases the firing rate of DA neurons in the VTA while simultaneously causing hyperpolarization of membrane potentials in DA neurons located in the substania nigra pars compacta (SNc), thus decreasing their firing rate [39]. Oxytocin release from the synaptic cleft causes both direct activation of DA neurons and indirect inhibition through local GABAergic neurons; however, the proportion of these two actions differs in the VTA and SNc depending on OXTR expression and circuit connectivity [39]. Another layer of complexity arises from the fact that the oxytocinergic circuitry works in a context-dependent manner. Optogenetic stimulation of hypothalamic oxytocinergic neurons directly reaching the amygdala in mice causes an increase in inhibitory postsynaptic current frequencies in a sub-population of neurons located in the amygdala as well as an unfreezing response (i.e., exerting anxiolysis), while inhibition of these neurons impairs fear-extinction [40]. Fear exposure in mice drives substantial plasticity in the hypothalamic-amygdala oxytocinergic circuitry, causing an elevation in glutamate levels in oxytocin axons terminating in the amygdala and mediating a shift from oxytocinergic to glutamate signaling [40]. This study demonstrates that established oxytocinergic circuits (e.g., hypothalamus-amygdala) are subjected to context-dependent, long-term plasticity reflected in modulated neurotransmission. Glutamatergic–oxytocinergic interaction also exists in the main hub of appetite regulation, namely the ARC (Figure 1). Glutamate releasing ARC neurons that express the OXTR and project to melanocortin 4 receptor (MC4R) expressing neurons in the PVN increase their excitatory post-synaptic potentials and induce rapid satiation when chemo- or optogenetically stimulated [41]. In vitro, oxytocin applied on synaptically isolated glutamatergic ARC neurons expressing the OXTR increases their firing rate, supporting a direct stimulatory effect of oxytocin on glutamatergic ARC neurons suppressing appetite [41]. Direct oxytocinergic neural pathways originating from the PVN and SON and reaching POMC OXTR expressing ARC neurons also induce satiation [27]. In this neurocircuitry, oxytocin directly stimulates the ARC POMC neurons by increasing their intracellular calcium levels [27]. In conclusion, the oxytocinergic neurocircuitry is characterized by intricate and varying interactions with multiple neural pathways that differ depending on the brain region and the contextual environment. Finally, oxytocin synthesized in magnocellular cells is carried by axonal transport to the posterior pituitary, where it is stored until release into the systemic circulation [42]. Peripheral circulating oxytocin affects multiple organs to exert metabolic effects [9], which are discussed subsequently. While the majority of oxytocin production occurs in the hypothalamus, oxytocin is also synthesized in peripheral organs, including the GI tract [43] and bone tissue [44], where it can exert metabolic effects in a paracrine/autocrine manner [45]. For instance, oxytocin produced by bone marrow osteoblasts in response to estrogen binds to the OXTR on osteoblasts to potentiate estrogen’s anabolic effects on bone [44], thus creating an autocrine feed-forward oxytocin/OXTR loop. Additionally, oxytocin synthesized in gastrointestinal enterocytes functions locally to reduce cellular endoplasmic reticulum stress, maintain the physical integrity of the gut epithelium, and induce anti-inflammatory effects [46]. It should be noted that animal models support species-specific sex differences in oxytocin synthesizing cells. For instance, while greater abundance of PVN oxytocin neurons was found in three types of female rodents compared with males, studies evaluating CNS sex differences in either oxytocin-synthesizing cells immunoreactivity or oxytocin mRNA expression could not detect such differences in eight other rodent species [47]. Whether these neurophysiological differences translate into functional sexually dimorphic actions of oxytocin is yet to be determined.

## 3. The Distribution of the Oxytocin Receptor in the Brain—Further Evidence Linking Oxytocin and Appetite Regulation

While our knowledge concerning the neuroanatomical and functional connectivity of the hypothalamic oxytocinergic neurons within the brain has increased significantly since the development of virus-based opto- and chemo-genetic physiological approaches [6,40,48], much more is known about the distribution of the OXTR in the brain. This knowledge is critical in constructing and testing theories relating to the effects of endogenous and exogenous oxytocin on appetite regulation and metabolism [49]. Oxytocin acts via a single oxytocin receptor which belongs to the seven-transmembrane G-protein-coupled receptor family and is coupled to phospholipase C through Gαq11 [50]. Binding of oxytocin to its receptor induces Ca^2+^ influx that depends on activation of phosphoinositide-3-kinase [6]. The end result of this process (neuronal activation or inhibition) depends on the type of α-protein subform of the receptor [6].

Seminal studies in animals and mostly rodents utilized histochemistry and immunohistochemistry methods to demonstrate the wide distribution of OXTR mRNA in subcortical and hypothalamic structures [6]. While some of these studies did not observe sex differences in OXTR binding density [47] or OXTR mRNA expression in areas critical for appetite regulation [51], others reported significantly greater OXTR mRNA expression and binding density in males compared with female rodents [47], suggesting that sexual dimorphism in the oxytocinergic system does not necessarily imply greater oxytocinergic activity in females. More recent human studies employed methods of monoclonal antibodies [52] as well as extensive gene mapping of the OXTR in human brains, showing a widespread distribution of the OXTR throughout the brain which was reproducible regardless of ethnicity and sex when tested with the latter method [53]. Interestingly, an exploratory analysis of the anatomical proximity between the OXTR and more than 20,000 other protein coding genes in the brain showed that genes regulating feeding (*NTSR2*) [54] and metabolism (*Glud1* and *Glud2*) [55] had robust overlap in brain expression patterns. Providing further confirmation of the link between the anatomical location of the OXTR and human eating behavior, a recent study found that OXTR brain expression patterns were strongly correlated with fMRI brain activation of areas categorized as anticipatory and appetitive [53]. In addition to the brain areas delineated above to which the oxytocin neurons send their projections (i.e., ARC, VTA, NAc, NST, amygdala), the oxytocin receptor is found in other brain areas [6,53] that are involved in the cognitive perception and reward value determination of food, thus potentially linking meal-related gut input and peripheral signals of energy bioavailability arriving at the hypothalamus with higher order brain input to regulate complex eating behaviors. These areas include the insular cortex [4], which functions as a multisensory gustatory neural hub [56]; the hippocampus [57], which plays an important role in goal-directed behavior, memory, and decision-making [58]; and the prefrontal cortex and anterior cingulate cortex, which are involved in attentional as well as cognitive control [34,59,60,61,62]. In conclusion, the neuroanatomical circuits of the oxytocin system provide a framework in which a mechanistic explanation for oxytocin participation in eating behavior can be formed.

## 4. The Oxytocin Receptor in the Periphery—Expression in Key Metabolic Organs

The distribution of the OXTR outside of the CNS also supports the role oxytocin plays in regulating key metabolic aspects closely related to feeding, body composition, bone health, glycemic control, and lipid metabolism [63]. Supporting the participation of oxytocin in the brain–gut neurohormonal axis, the OXTR can be found along the gastrointestinal (GI) tract in myenteric and submucous ganglia where it has a role in regulating GI motility, promoting the development of enteric neurons, and opposing intestinal inflammation [64,65]. Importantly, the OXTR is also expressed on afferent vagal nerves which transmit visceral input to the brain [65]. The majority of these fibers reach the NTS mentioned above, which is densely innervated by oxytocin PVN afferent neurons and from which axonal projections to PVN oxytocinergic neurons exist [66], thus forming a circuit regulating GI functioning [67]. The afferent oxytocinergic vagal signaling route has imperative importance in mediating CNS effects of peripherally administered oxytocin [68].

The expression of the OXTR in musculoskeletal and adipose tissues support its involvement in the regulation of body composition; namely, the balance between bone, adipose, and muscle tissues [69]. The OXTR is present on human myoblasts [70] and muscle stem cells [71,72], where it promotes skeletal muscle regeneration and protects against age-related sarcopenia by directly acting on muscle stem cells to induce myogenesis [73]. In vitro, the OXTR has also been shown to inhibit the differentiation of mesenchymal stem cells to adipocytes while promoting cardiomyogenesis and osteogenesis [71,72]. From a metabolic standpoint, the suggested dual effects of oxytocin on mesenchymal stem cells of maintaining viable muscle tissue and minimizing adipose tissue generation could have beneficial metabolic implications relating to anti-inflammatory effects, reduced insulin resistance, and improved glycemic control [74]. Within the musculoskeletal system, the OXTR can also be found on osteoblasts and fully differentiated osteoclasts and their precursors, where it exerts overall anabolic effects [75,76]. More specifically, the presence of the OXTR on osteoblasts is necessary to promote the development of a mineralizing phenotype [77] and to mediate the anabolic effects of estrogen on bone through osteoblast maturation [76]. While oxytocin promotes osteoclastogenesis from hematopoietic stem cell precursors, it also inhibits bone resorption by mature osteoclasts [78]. In addition to its pivotal role in lactation and parturition, oxytocin may also be involved in the regulation of maternal skeletal homeostasis during pregnancy and breastfeeding, both characterized by major alterations in calcium demands [77,79].

**Figure 2:**
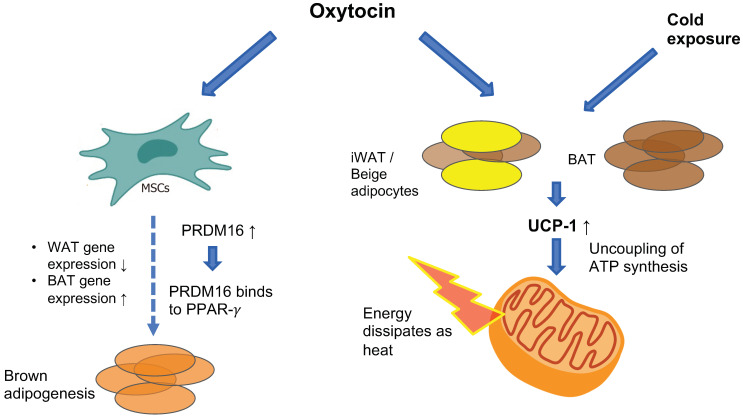
Proposed mechanism for the effects of oxytocin on thermogenesis and brown adipogenesis. Oxytocin may be an adipocyte browning inducer—in vitro treatment of pluripotent mesenchymal stem cells with oxytocin suppresses gene expression of white-adipose selective markers (*Nnmt* and *Retn*) while increasing expression of thermogenic genes (e.g., *UCP-1*, *Cidea*, *Ppargc1a*, *Dio2*, and *Elovl3*). Oxytocin also up-regulates PRDM16—a transcription factor that promotes brown adipose tissue (BAT) formation by binding to peroxisome-proliferator activated receptor-gamma coactivator 1 alpha (PPAR-gamma) and inducing brown adipogenesis from progenitor myoblasts. PRDM16 also suppressed white-adipocytes selective genes in BAT. Oxytocin also increases the expression of uncoupling protein-1 (UCP-1) in BAT and inguinal white adipose tissue (iWAT)/beige adipocytes. UCP-1 is a mitochondrial membranous protein that is uniquely expressed in brown adipocytes and has a pivotal role in BAT adaptive thermogenesis. UCP-1 promotes proton translocation through the inner membrane of brown adipocyte mitochondria, thus diverting respiration from ATP synthesis to energy dissipation in the form of heat. Specific cues (e.g., cold exposure) also increase UCP-1 expression and thermogenesis. In vivo, oxytocin enhances the expression of UCP-1 in both mice and rat BAT and iWAT, thus promoting thermogenesis.

The OXTR is highly expressed in white adipose tissue (WAT) [80] and to a lesser extent in brown adipose tissue (BAT) [81], where it promotes increased energy expenditure by stimulating BAT thermogenesis and promoting WAT browning (see Figure 2). Chronic intracerebroventricular (ICV) administration of oxytocin to diet-induced obese male rats and mice increases BAT temperature and this effect is abolished when animals receive pre-treatment with an OXTR antagonist [82,83]. Similarly, chronic subcutaneous oxytocin treatment in male mice exposed to cold temperatures increases core body temperature [81]. The molecular mechanism through which oxytocin induces these changes has been revealed recently. Oxytocin is an adipocyte browning inducer—in vitro treatment of pluripotent C3H10T1/2 stem cells with oxytocin suppresses the expression of white-adipose selective markers (*Nnmt* and *Retn*) while increasing the expression of thermogenic genes (e.g., *Cidea*, *Ppargc1a*, *Dio2* and *Elovl3*) which are considered as brown adipocyte-selective markers [81]. Among these genes is uncoupling protein-1 (*UCP-1*), a mitochondrial membranous protein that is uniquely expressed in brown adipocytes and has a pivotal role in BAT adaptive thermogenesis. UCP-1 promotes proton translocation through the inner membrane of brown adipocyte mitochondria, thus diverting respiration from ATP synthesis to energy dissipation in the form of heat [84]. UCP-1 is also present in beige adipocytes, which reside within the WAT depot, mostly in inguinal WAT (iWAT) in rodents. Beige adipose tissue also participates in thermogenesis in response to specific cues (e.g., cold exposure or adrenergic signaling) [85]. Exposure of mice to cold temperatures induces an increase in hypothalamic oxytocin and adipose-tissue OXTR expression [81], and ablation of hypothalamic oxytocin neurons results in impaired thermogenesis in response to cold exposure which is rescued with oxytocin treatment [86]. In vivo, oxytocin enhances the expression of UCP-1 in both mice and rat BAT and iWAT, thus promoting thermogenesis [81,83]. Oxytocin also up-regulates *PRDM16*, which is a transcription factor that promotes BAT formation by binding to peroxisome-proliferator-activated receptor-gamma coactivator 1 alpha (PPAR-gamma, or Pgc-1 alpha) and inducing brown adipogenesis from progenitor myoblasts in C3H10T1/2 stem cells [87]. PRDM16 has also been shown to suppress white-adipocyte selective genes in BAT [88]. In *PRDM16*-deficient mice, oxytocin-induced increase in UCP-1 was significantly attenuated [81], suggesting that oxytocin-induced adipocytes browning is mediated by PRDM16.

In contrast to the study discussed above by Yuan et al., showing in vivo and in vitro adipocytes browning under oxytocin treatment [81], Sun et al. found that in vitro, oxytocin had inhibitory effects on white-to-beige transition gene programming. More specifically, incubation of adipocytes derived from precursor 3T3.L1 cells with oxytocin resulted in reduced expression of “beiging” genes (i.e., *Cox8b*, *Cebpb* and *Cidea*). The authors of this study postulated that this effect could conserve energy in the setting of increased satiety due to the central effects of oxytocin on appetite [78]. The discrepancy between studies could be explained by the different methodologies used in the two studies. While Yuan et al. used C3H10T1/2 pluripotent stem cells, which are morphologically and functionally similar to mesenchymal stem cells, Sun et al. used preadipocyte 3T3.L1 cell lines. In the state of obesity, an increase in the size and number of adipocytes occurs due to recruitment of pluripotent and mesenchymal stem cells that are located in the vascular stroma of the adipose tissue. These stem cells have the potential to undergo differentiation and commitment to either adipose, muscle, bone, or cartilage lineages, depending on the triggering cues in their environment [89]. While C3H10T1/2 are multipotent stem cells, 3T3.L1 cell lines represent preadipocytes, meaning that these cells have already undergone commitment to the adipose lineage [90,91]. It is possible that oxytocin exerts different in vitro effects on these two cell populations; however, more research is required to clarify this question. It is also important to note that clonal studies have inherent limitations as described in a recent comprehensive review on adipose tissue plasticity and heterogeneity [92]. These limitations include the characteristics of in vitro studies which oversimplify the intricate environment in which native cells reside, the over-representation of highly proliferative cells in the clone, and the fact that cells derived from a single cell line potentially lack intercellular communications with other cells that reside within the native tissue [92]. To summarize, while Sun et al. and Yuan et al. report contradicting evidence regarding oxytocin’s in vitro effects on genes promoting adipose-tissue browning, several studies have shown that oxytocin’s observed increase in thermogenesis is accompanied by enhanced expression of UCP-1 in WAT, suggesting that it induces WAT browning in vivo [81,83,93]. Additional research is needed to more clearly define the effects of oxytocin on adipose tissue gene programming.

OXTR expression on adipocytes is increased in the state of obesity [94] and may have a role in counteracting the inflammatory response associated with excess weight. Adipose tissue expansion resulting from weight gain is associated with infiltration of the tissue by macrophages and the production of pro-inflammatory cytokines [95]. This low-grade, chronic inflammation is critically involved in the pathogenesis of obesity-related metabolic syndrome [96]. When oxytocin was subcutaneously infused to db/db obese mice, it directly suppressed visceral adipose tissue inflammation, as reflected by decreased mRNA expression of IL-6 and TNF-alpha and increased levels of anti-inflammatory, protective adipokines [97]. These changes were seen prior to weight loss, suggesting a direct anti-inflammatory effect of oxytocin on adipose tissue. Finally, the OXTR is also directly involved in stimulating lipolysis and fatty acid beta oxidation [98]. Taken together, these findings support the favorable participation of the OXTR in fat metabolism.

The OXTR is expressed on beta and alpha cells in the pancreatic islets of Langerhans in rodents [99] and humans [100] and is involved in stimulating beta cell proliferation while protecting against apoptosis [100,101]. Whether oxytocin and the OXTR directly regulate insulin release from the pancreas is still under investigation, with some studies showing improved glycemic control following oxytocin administration in rodents [98,102,103] and others demonstrating negative effects in leptin-deficient ob/ob mice [104] and leptin-resistant obese Zucker-fatty rats [105]. A more in-depth discussion of the involvement of oxytocin in glucose homeostasis is provided later in the review.

The OXTR can also be found abundantly in cardiomyocytes, where it exerts cardioprotective effects through different signaling pathways. These include deceleration of atherosclerosis, protection from myocardial metabolic and inflammatory injury, as well as post-ischemia repair [106,107].

## 5. Data from Animal Models-Paving the Path to Oxytocin Translational Research

In addition to the accumulating data demonstrating the incorporation of oxytocin and its receptor in key brain areas and organs mediating energy balance and metabolism, animal studies provide further support to the notion that oxytocin drives energy homeostasis in an advantageous manner [10,12,13,108]. These studies examined the effects of oxytocin and OXTR agonists and antagonists and utilized knockout oxytocin and OXTR mouse models. Here, we synthesize key findings derived from animal studies that established the therapeutic potential of oxytocin and laid the groundwork for translational research in humans. A comprehensive, detailed review of oxytocin research in animal models is beyond the scope of the current review and has been published before [10,12,108].

### 5.1. Oxytocin Administration in Animal Studies

Initial foundational studies conducted in the early 1990s showed that ICV [109,110,111] and intraperitoneal [109,110] administration of OXTR agonists in rodents resulted in acute food intake reduction which was abolished when animals were pretreated with an OXTR antagonist. The administration of an OXTR antagonist by itself increased food consumption in food-deprived animals in some studies [109,110,112,113] but not in others [111,114]. Of note, the vast majority of these studies have been conducted in male animals and whether oxytocin administration suppresses food intake to the same extent in female rodents is yet to be determined. A recent study has shown that while ICV oxytocin significantly reduced chow intake in both male and randomly cycling female rodents, the effect was significantly more pronounced in males [51]. Moreover, in female rodents, oxytocin attenuated food intake throughout the menstrual cycle, with the exception of the proestrus phase [51], suggesting that sex hormones interact with oxytocin to modulate food intake. Over the years, multiple studies conducted in different animal models recapitulated the anorexigenic effects of acute oxytocin treatment, supporting its role in signaling satiation [115]. These studies showed that in addition to inducing acute hypophagia in normal-weight rodents, long-term oxytocin treatment attenuated weight gain or reduced body mass in diet-induced obese rodents [98,102,103,116,117] and non-human primates [118] as well as genetically manipulated obese animals, including leptin-resistant db/db obese diabetic mice [93,119] and leptin-resistant Zucker-fatty rats [105,120]. Importantly, oxytocin-induced weight loss in obese animals occurred with preferential reduction in fat mass while preserving the metabolically healthy lean mass and with evidence for increased lipolysis, enhanced fat oxidation, and reduction in adipocyte size [98,103,105,121,122]. The reduction in total body mass seen under chronic oxytocin treatment could not be explained by the anorexigenic properties of oxytocin alone, since long-term oxytocin administration has been associated with diminished effects on caloric intake while the effects on body mass were sustained [12,98,103]. These findings are consistent with the results of a recent comprehensive quantitative meta-analysis examining the effects of oxytocin treatment on food intake in animals. It was found that a single oxytocin dose given via peripheral or central administration resulted in subsequent reduced caloric intake; however, when trials lasting longer than two weeks were analyzed, final-day caloric intake did not differ between oxytocin and placebo treatment [123]. The reduction in body weight seen under chronic oxytocin treatment in the absence of hypophagia has been attributed to oxytocin-induced increase in energy expenditure seen in diet-induced obese non-human primates [118] and rodents [116,124]. It has also been suggested that chronic oxytocin treatment could counterbalance the expected decrease in energy expenditure that results from weight loss [122], potentially via oxytocin-induced increase in BAT thermogenesis [12,82]. In a recent study, central administration of oxytocin over 28 days to diet-induced obese mice resulted in weight loss associated with preferential reduction in white adipose tissue size and an increase in brown adipose tissue thermogenesis, thus supporting this hypothesis [83]. Whether chronic oxytocin treatment induces sex-specific effects relating to energy balance is currently unknown due to a dearth of studies conducted with female animals and given the conflicting results seen in the few investigations conducted to date [12,123]. From a glucose metabolism standpoint, chronic oxytocin administration in animal models of diet-induced obesity and diabetes has resulted in improvement of glucose tolerance and insulin resistance in the majority of studies [81,98,103,116], with one study showing worsening of basal glycemia, glucose tolerance, and insulin sensitivity in extremely obese and diabetic (ob/ob) mice [104]. The authors of the latter study hypothesized that the deterioration in glycemic control occurred due to enhanced hepatic gluconeogenesis associated with oxytocin-induced increase in corticosterone levels (seen only in ob/ob, but not in lean mice) as well as the severe hyperinsulinemia seen in ob/ob mice, resulting in amplified burden on the pancreatic beta cells and thus an inability to meet demands for increased insulin secretion [104].

### 5.2. Oxytocin and Oxytocin Receptor Gene Knockout Experiments

In parallel with the line of research employing oxytocin administration in animals, oxytocin and OXTR gene knockout studies have been instrumental in further elucidating the different roles oxytocin plays in the coordination of energy balance. Initial studies have shown that mice homozygous for deletions of either oxytocin [125] or the OXTR [126] developed late-onset obesity which was not associated with hyperphagia. In addition to having excess weight, these mice displayed increased proportion of fat mass, hyperlipidemia, decreased insulin sensitivity, impaired thermogenesis [97,125,126,127], premature sarcopenia [73], as well as osteoporosis due to impaired bone formation [76,77]. Metabolic perturbations, including decreased energy expenditure and/or decreased muscle tonicity, are thought to drive obesity in normophagic oxytocin/OXTR-deficient mice. More specifically, it was recently hypothesized that the lack of oxytocin potentiating actions on slow-twitch skeletal muscles in oxytocin/OXTR knockout mice could lead to progressive intramuscular adipose tissue accumulation. Coupled with sarcopenia, the gradual decrease in muscle strength and tonicity could have contributed to the attenuated energy expenditure and the late-onset, normophagic phenotype of these knockout mice [128]. Impaired thermogenesis has been clearly established to occur in oxytocin/OXTR-deficient rodents and specifically in response to cold exposure [86,97,127,129]. Perturbated heat production and therefore reduced caloric burn provide an additional mechanism to explain the phenotype of obesity in the absence of increased food intake.

Other genetic conditions that result in an insult to PVN oxytocin expressing neurons also result in a disruption of energy homeostasis. Haploinsufficiency of single-minded 1 gene (*SIM1*), a transcription factor involved in the development of the PVN and SON of the hypothalamus, results in reduced expression of oxytocin in the PVN in mice and is associated with hyperphagia, decreased energy expenditure, and obesity that improve with oxytocin administration [130,131]. Prader–Willi syndrome (PWS), which is also associated with a depletion of hypothalamic oxytocin producing neurons [132] as well as a reduction in the peripheral expression of the OXTR in humans [133], presents with hyperphagia, lack of satiety, obesity, hypotonia, and unfavorable body composition [134]. An in-depth discussion of clinical conditions in humans involving hypothalamic and associated oxytocinergic insult resulting in abnormal energy homeostasis and eating behavior is provided later in the review.

## 6. Observational Studies in Humans-Relation of Oxytocin to Food Intake, Weight Status and Metabolism

### 6.1. Characterization of Endogenous Oxytocin Levels in Humans—Important Technical Aspects and Challenges

In light of the accumulating data from animal studies establishing oxytocin as an anorexigenic hormone with favorable metabolic effects, human research dedicated to the relationship between endogenous oxytocin status and metabolic health and disease has accelerated over the past two decades [9,12]. These studies have been instrumental in comparing oxytocin levels between different populations within the same study or in response to stimuli. However, consolidation of these data has faced significant hurdles related mostly to the following: (a) technical challenges associated with oxytocin measurement [135], (b) inherent sex differences in oxytocin physiology [136], and (c) a lack of complete understanding of the relation between peripheral oxytocin levels and CNS oxytocin activity [137,138,139].

### 6.2. Oxytocin Measurement in Humans

The oxytocin molecule has a very short half-life (3–6 min in the periphery and 19 min in the CNS) [140,141]. It is quickly degraded by multiple peripheral and brain aminopeptidases [142,143,144] and its baseline circulating levels in non-pregnant/non-lactating individuals are very low (<8 pg/mL) [145]. In addition to these inherent characteristics, the major challenge relating to the research of endogenous oxytocin levels is the lack of standardized measurement techniques. First, different forms of oxytocin exist in plasma (extended, bound, and fragments of oxytocin). Following synthesis of the inactive oxytocin prohormone, it is first converted to precursors or extended forms of oxytocin consisting of up to twelve amino acids [16,146], which are subsequently cleaved to the nine-amino acid molecule of oxytocin [147]. The extended forms of oxytocin have been shown to have biological activity; e.g., in mice, both oxytocin and its precursor have a critical role in cardiac differentiation of embryonic stem cells [148]. The oxytocin molecule itself can be found in the circulation bound to multiple proteins and it can be further processed by cleavage to fragments of oxytocin [4]. Cleaved forms of oxytocin also possess biological activity, e.g., they have been shown to affect pain threshold through interaction with opioid receptors and fragments of oxytocin may interact with alpha 2-adrenoreceptors to regulate blood pressure [149,150,151]. Importantly, the detection of oxytocin’s different forms differs substantially depending on the approach used for preparation of the sample and measurement of oxytocin. Whether extraction/filtration methods should be used prior to measuring oxytocin levels is controversial. These techniques employ pre-treatment of the sample with a solvent to induce precipitation of interfering proteins and isolation of the soluble oxytocin molecule. While some argue that pre-processing of the sample with extraction/filtration is required to remove interfering components [138], a limitation of using these methods relates to the potential removal of biologically active, protein-bound oxytocin molecules [135]. It should be noted that extracted versus non-extracted samples lead to oxytocin levels that are different in magnitude (e.g., 1.8 pg/mL vs. 358 pg/mL in extracted vs. non-extracted sample, respectively) [138,152,153], thus necessitating caution when integrating data from studies employing and not employing this method. The use of substantially different approaches throughout the years to measure the oxytocin molecule itself further complicates our understanding of oxytocin physiology [135]. The two main methods used to measure oxytocin include liquid chromatography mass spectrometry (LCMS) and immunoassays that employ binding of oxytocin to an antibody (i.e., Radioimmunoassay (RIA) and Enzyme Linked-Immunosorbent Assays (EIA)). The various available immunoassay techniques have been shown to produce substantially different results [138,152], potentially due to lack of standardized antibodies, cross-reactivity of antibodies with extended forms and fragments of oxytocin as well as non-oxytocin components, and heterophilic interference [135]. Compared with immunoassays, LCMS is generally considered a more specific and accurate method for the detection of molecules. However, fewer studies have been conducted to assess oxytocin levels using LCMS methods (two recent examples include [145,154]), with some showing poor reproducibility and precision [155]. To date, the development of a single technique producing specific, sensitive, reliable, and reproducible oxytocin levels is still in process. Still, the methods available today have been useful in assessing alterations in oxytocin levels in response to stimuli, comparing oxytocin concentrations in different populations within the same study, and analyzing the association of oxytocin levels to clinical and biochemical parameters of interest. A unifying approach to the measurement of oxytocin will advance our ability to investigate the role of oxytocin in energy balance and metabolism.

### 6.3. Sex Differences in the Oxytocin System

Potential sex differences in the physiology of oxytocin represent another important factor to take into consideration when consolidating data from different studies aimed at characterizing endogenous oxytocin levels. Human sex differences in central and peripheral oxytocin and OXTR expression is an understudied research area [136]. Early studies did not show sex differences in the number or size of the hypothalamic oxytocin neurons [156,157,158]. However, higher levels of oxytocin in the cerebrospinal fluid (CSF) were found in women compared with men [159], suggesting that the morphological characteristics of the PVN and SON oxytocin neurons do not necessarily reflect the release of oxytocin in the CNS. More recent studies of peripheral oxytocin levels in women compared with men have shown inconsistent results, with some finding higher levels in females [160,161], another study reporting elevated oxytocin levels in males [162], and several studies showing no sex differences in circulating oxytocin [163,164,165]. These inconsistencies could be related to studying heterogenous populations and to the fact that peripheral oxytocin levels are also affected by a variety of factors which were not systematically controlled for, including excess weight and associated metabolic disease [166], aging [167], use of oral contraceptive pills [168], and chronic diseases [169]. While sex differences in circulating oxytocin are not well established, there is evidence showing that intranasal (IN) oxytocin induces divergent, sex-specific behaviors and fMRI patterns in humans performing socio-emotional [170,171] and social reward [172] tasks. These observations, together with animal studies showing that estrogens [173,174] and androgens [175,176] can modulate PVN oxytocin neuron mRNA expression, support the hypothesis of sex differences in endogenous oxytocin status. Refinement of measurement techniques and adjustment for confounding factors affecting oxytocin physiology are needed to further research this question.

### 6.4. The Relation between Peripheral Oxytocin Levels and Central Oxytocin Activity

To date, multiple studies have looked at the association between peripheral oxytocin levels and metabolic outcomes of interest as a proxy for oxytocin central effects on appetite regulation, energy expenditure, and metabolism. It is unclear, however, if peripheral oxytocin levels in humans accurately reflect oxytocin central activity. As discussed previously, oxytocin is released within the brain and to the peripheral circulation via separate, independent pathways, each associated with a different time scale of action. More specifically, oxytocin release from the magnocellular PVN and SON neurons to the periphery via the posterior pituitary occurs through axonal projections and is instantaneous while magnocellular somato-dendritic secretion and diffusion of oxytocin within the brain is slower and occurs over minutes [19]. The existence of these two mechanisms suggests that at least some oxytocin central activity (i.e., the activity mediated by dendritic release) is not temporally synchronized with oxytocin peripheral activity. In contrast, centrally injected alpha-melanocyte stimulating hormone (alpha-MSH), which has a pivotal role in inducing satiety, acts on SON oxytocin receptors expressing alpha-MSH receptors to directly stimulate central oxytocin dendritic release while simultaneously suppressing electrical activity of oxytocin cells and thus the secretion of oxytocin into the systemic circulation [67]. Very recently, a meticulous description of oxytocin connectivity has been achieved in rodents using state-of-the-art engineered viral tracers [26]. This study showed that a subset of magnocellular oxytocin neurons collaterally and simultaneously projects to both the posterior pituitary and additional brain areas involved in reward and reinforcement-based learning behaviors (e.g., the amygdala, dorsal striatum, lateral septum, and NAc) [26] (Figure 1). Chemogenetic activation of these neurons provoked central oxytocin release to regulate social interactions and locomotion concurrently with elevation in peripheral oxytocin release [26]. In conclusion, the neurocircuitry of oxytocin supports both coordinated and non-coordinated central and peripheral activity.

The correlation between plasma and CSF oxytocin levels provides another surrogate for the synchrony between CNS and peripheral oxytocin actions. However, studies conducted to research this question have been inconsistent. While two studies found a correlation between plasma and CSF oxytocin levels in adults presenting with acute headaches [177] and children and adults undergoing indicated lumbar puncture (LP; e.g., due to meningitis, pseudotumor cerebri, new diagnosis of leukemia) [178], others did not demonstrate such a relationship in non-neurological adult patients undergoing spinal anesthesia for minor surgical procedures [179], in critically ill patients with neurological disorders [139], and in nonpregnant or pregnant females undergoing labor or elective c-section [180,181]. In addition, a correlation between central and peripheral oxytocin levels was not found in neurosurgical patients requiring external ventricular drain who were not exposed to painful/stressful stimuli associated with the LP procedure [137]. These inconsistencies between studies could be related to the heterogeneity in studies’ participants, the different sampling conditions [182] and the variability in the presence of acute stressful stimuli at the time of testing [183,184]. The lack of correlation between CSF and plasma oxytocin levels could further support the existence of uncoordinated central-peripheral oxytocin actions; however, more research is needed to elucidate this question. Finally, it is noteworthy that in critically ill neurosurgical patients, salivary oxytocin correlated better with CSF compared with plasma oxytocin levels [137,139]. It will be important to further investigate the relation between plasma, salivary, and CSF oxytocin status in different populations.

### 6.5. Alterations of Endogenous Oxytocin Levels in the Setting of Obesity and Metabolic Syndrome

Previous studies investigating the association between circulating oxytocin, obesity, and adverse metabolic outcomes of interest have shown equivocal results.

In a cohort of 59 women across the weight spectrum (18–45 years of age), a positive association was found between overnight, frequently sampled oxytocin levels and BMI as well as total and visceral fat mass [185]. This study included amenorrheic patients with anorexia nervosa (a state of energy deficiency), eumenorrheic healthy controls, and eumenorrheic women with obesity (a state of surplus energy). Based on the positive association between oxytocin and BMI as well as fat mass, the authors concluded that oxytocin may be a biomarker of energy availability [185]. Similarly, in a small pilot study examining 18 healthy women across the weight spectrum with a wide range of ages (18–85 years), a positive correlation between BMI and plasma oxytocin levels was observed and remained significant after controlling for age [186], with higher levels of oxytocin in women defined as food-addicted according to the Yale Food Addiction Scale (YFAS). In contrast to the two previous studies, another study designed to investigate the association between food addiction (per the YFAS questionnaire) and oxytocin status in 80 women with obesity and with a wide range of ages (18–60 years) revealed inverse relationships. This study found lower oxytocin levels in women with food addiction compared to those without food addiction as well as a negative correlation between oxytocin levels and body weight [187]. Similarly, in a cohort of 311 women ages 18 to 45 years, those with obesity had lower oxytocin concentration compared with healthy-weight females [188]. The same study found that oxytocin levels were inversely associated with LDL-cholesterol and positively associated with HDL-cholesterol [188]. The discrepancies between these studies limited to female participants could be related to different measurement techniques and lack of control for obesity-associated comorbidities which could have an effect on oxytocin levels (discussed subsequently). Differences in hormonal status (e.g., menopause, the menstrual cycle, and use of oral contraceptive pills (OCPs)) are another potential source of the conflicting results. While some of these studies included participants taking OCPs, which could increase circulating oxytocin levels [189], one study excluded such subjects [185] and in another study, OCP use was not addressed [188]. Additionally, in healthy women, circulating oxytocin levels are lower in the early to mid-follicular phase of the menstrual cycle compared with the levels measured at other menstrual cycle phases [190]. In three of the studies mentioned above, oxytocin levels were taken during the follicular phase of the menstrual cycle [185,186,187] but at different times, and one of these studies did not account for the differences in oxytocin levels during the menstrual cycle [188]. In summary, in order to fully understand the relationships between circulating oxytocin levels and weight status, future studies should account for the potential hormonal differences between female participants.

Since the post-menopausal years are associated with an increased risk for metabolic syndrome independently of age [191] and given the fact that oxytocin central signaling is modulated by estrogens [174], a few studies investigated oxytocin status by pre/post-menopausal stratification. In a cross-sectional study examining circulating oxytocin levels in pre- vs. post-menopausal women (*n* = 106), a stepwise multivariable regression analysis showed that both BMI and menopause were independent predictors of oxytocin levels, explaining 32% of the variability in oxytocin concentration [192]. In this study, plasma circulating oxytocin levels were significantly higher in premenopausal compared with postmenopausal women and on average 3.5-fold higher in healthy-weight subjects compared with those with obesity. This difference in oxytocin levels between healthy weight participants and those with obesity was greater in postmenopausal women, when oxytocin was detected at the lowest concentrations [192]. Across the entire cohort, oxytocin levels were inversely associated with BMI, visceral and whole-body fat mass, insulin resistance, and dyslipidemia [192]. Two additional studies examined only postmenopausal women and found different relations between BMI and oxytocin. In the first study, no association was found between oxytocin levels and BMI in postmenopausal women [193]. Conversely, in a larger cohort of postmenopausal women (*n* = 1097), a positive correlation between oxytocin levels and BMI was observed [194]. Taken together, studies limited to female participants evaluated across the lifespan or in relation to their reproductive/menopausal status show conflicting results in regard to the association of oxytocin to BMI and metabolic health.

Several previous studies aimed to investigate the relation of oxytocin to adiposity and metabolic health in both men and women. In a recent cross-sectional study evaluating 721 men and women with a mean age of 47 years, oxytocin levels were not only positively correlated with excess weight, but a significant positive correlation was also found between circulating oxytocin and indices of metabolic syndrome, including impaired glucose tolerance, homeostasis model assessment-estimated insulin resistance (HOMA-IR), triglyceride, LDL cholesterol, total cholesterol, and central obesity [166]. The correlation between oxytocin and triglycerides as well as HOMA-IR remained significant after controlling for age, sex, and BMI, suggesting that elevated oxytocin is associated with metabolic perturbations independent of adiposity status. Of note, patients with T2DM were excluded from the correlation analysis in this study. Similarly, in another study examining non-diabetic individuals, positive significant correlations were observed between oxytocin and a number of glycemic indices, including fasting glucose and insulin levels and HOMA-IR after adjustment for age, gender, and BMI [195]. Consistent with these studies, in a similar study design involving 504 male participants ages 50–85 years, higher oxytocin levels were associated with greater body weight, increased incidence of diabetes, increased proportion of total and central fat mass, elevated triglycerides, lower HDL cholesterol, and higher odds of having metabolic syndrome [196].

In contrast to these studies, opposite relations between oxytocin and metabolic disease were found by Qian et al. [197]. In this investigation, 176 participants were equally stratified based on their weight and glycemic control statuses. Eighty-eight participants were healthy-weight men and women, with half demonstrating normal results on a glucose tolerance test (NGT), and the rest were recently diagnosed with type 2 diabetes (T2DM). The other additional 88 participants had obesity; half of those were also newly diagnosed with T2DM and the rest had obesity with NGT [197]. In this study and in contrast to the others mentioned above [166,195,196], oxytocin levels were inversely correlated with BMI as well as additional obesity-related adverse metabolic parameters (e.g., HbA1c, glucose and fasting insulin levels, total cholesterol, triglycerides, LDL cholesterol, and HOMA-IR) [197]. In binary logistic regression analyses, oxytocin concentration was significantly associated with T2DM after adjustment for BMI, age, and sex, suggesting that the state of diabetes affected oxytocin independently of weight status [197]. Consistent with this notion, individuals with newly diagnosed T2DM, with and without obesity, exhibited similar oxytocin levels which were lower than those of NGT subjects, regardless of their weight status [197]. The association of lower oxytocin levels with prediabetes/T2DM is consistent with a number of previous reported results [198,199,200]. It is possible that the inclusion of newly diagnosed and therefore uncontrolled diabetic patients together with individuals with normal glycemic control in the healthy-weight and obesity groups biased the evaluation of oxytocin levels by BMI status. This stratification could potentially explain the discrepancies in the relationship between oxytocin and BMI in this study compared with others.

Alteration of oxytocin following weight reduction has also been the subject of research, producing conflicting results. In an early, small study conducted in the late 1980s evaluating 23 male participants, plasma oxytocin levels did not differ between subjects with and without obesity and were unchanged following weight reduction [201]. In another investigation performed in the late 1980s, oxytocin levels were significantly elevated in men and women with obesity compared with healthy-weight individuals [202]. In this study, circulating oxytocin decreased significantly six months following gastric-banding bariatric surgery associated with weight reduction, and oxytocin levels remained elevated compared with those of lean subjects [202]. In a more recent investigation looking at a cohort of 109 men and women with a wide range of BMIs and without diabetes, oxytocin levels were similar in all BMI categories (healthy weight, overweight, class I and II obesity), with the exception of individuals with BMI > 40 kg/m^2^ who had significantly elevated oxytocin levels [203]. Across all study participants, a positive correlation was found between oxytocin levels and BMI as well as insulin levels and HOMA-IR. In 12 individuals with morbid obesity who underwent Roux en-Y gastric bypass surgery and subsequent major weight reduction, oxytocin levels were unchanged one year following the procedure [203]. Finally, oxytocin concentration was analyzed in 62 women with obesity who were randomly assigned to receive probiotics or a placebo in addition to a restricted calorie diet for 12 weeks [204]. Compared with women who received the placebo, women under probiotic treatment experienced significantly greater reduction in BMI, a more pronounced improvement in eating behavior, and a greater increase in oxytocin levels. Previous studies have shown that supplementation with *Lactobacillus reuteri* (which was included in the probiotics given in this study) can increase oxytocin levels [205]. Hence, the authors of this study hypothesized that the increase in oxytocin levels induced by probiotic treatment contributed to the improvement in weight management [204]. Taken together, it can be concluded that studies show inconsistent changes in oxytocin levels following weight reduction, which could be related the different hormonal/metabolic changes associated with each method of bariatric surgery [206] and the independent mechanisms related to the interaction of probiotic treatment with oxytocin [205].

In conclusion, studies conducted to date do not provide a clear picture of the relationship between oxytocin levels and BMI, metabolic syndrome, and weight reduction. Elevated oxytocin levels in the setting of obesity could indicate that oxytocin is a biomarker of energy availability and may represent a compensating mechanism given the anorexigenic properties of this hormone. Inverse relationships between oxytocin concentration and BMI could be related to poor glycemic control and could hypothetically contribute to the development of obesity. The major differences between studies could stem from various clinical and technical reasons—including the presence of diabetes, differences in hormonal status, age, gender, microbiome, and the issues related to oxytocin measurement (e.g., sampling and storage conditions, preparation of samples, and measurement techniques). Well-designed prospective studies examining alterations of oxytocin concentrations following weight reduction would also be important to conduct. Finally, a unifying approach to oxytocin measurement and an in-depth understanding of the clinical factors affecting oxytocin levels will be critical in advancing our knowledge.

### 6.6. The Relation of Peripheral Oxytocin Levels to Bone Health

Cross-sectional studies support a positive association between circulating oxytocin levels and parameters of favorable bone health in different populations.

Oxytocin levels were significantly lower in postmenopausal women with severe osteoporosis compared with healthy controls, and positively correlated with both lumbar spine and femoral neck bone mineral density (BMD) across the entire cohort independent of other characteristics which may affect bone health (e.g., age, BMI, ultra-sensitive estradiol levels, steroid, or hormonal replacement treatment) [193]. In a larger cohort of postmenopausal women with osteoporosis (16%), osteopenia (48%), and normal BMD (36%), a positive correlation was observed between oxytocin levels and favorable spine and total hip BMD [194]. In this study, a separate analysis was performed in women with undetectable estradiol levels (almost 90% of the study population), revealing a significant positive correlation between oxytocin levels and total hip BMD [194]. Oxytocin levels were not associated with a history of fractures [194]. The relation between bone health and oxytocin has also been studied in pre-menopausal women. In a cohort of 59 women across the weight spectrum (18–45 years of age), a positive association was found between pooled oxytocin levels measured overnight and spine and hip BMD z scores [185]. Nocturnal oxytocin levels were also lower in young amenorrheic women with anorexia nervosa compared with healthy controls and associated with decreased BMD at the spine [207]. Young, amenorrheic athletes also exhibited lower overnight secretion of oxytocin which was associated with impaired bone microarchitecture at the distal tibia and radius [208]. The relation of peripheral oxytocin to bone health was also studied in men with central diabetes insipidus (CDI), a condition caused by a deficiency of the posterior pituitary hormone vasopressin which shares anatomical pathways with the oxytocin system. In individuals with CDI, lower fasting plasma oxytocin levels were associated with lower bone mineral density and unfavorable bone hip geometry [209]. In contrast to the studies discussed above, in men aged 50 years and older recruited for the prospective MINOS of osteoporosis and of its determinants, serum oxytocin levels were not associated with BMD at any site; however, a negative, weak association was found with fracture risk [210], requiring further investigation of this observation in larger cohorts.

Taken together, menopause-associated osteopenia/osteoporosis seem to be related to hypo-oxytocinergic state, supporting the involvement of oxytocin in the pathophysiology of postmenopausal osteoporosis. Additionally, lower oxytocin levels in premenopausal women, patients with anorexia nervosa, and men with CDI are also associated with lower BMD. Further research is needed to study the association between oxytocin levels and fracture risk in men and women. Whether oxytocin can be used as a therapeutic agent to improve bone health in these conditions is an important area of future research.

## 7. Interventional Studies of Oxytocin Administration in Humans

### 7.1. Effects of Oxytocin Administration on Eating Behavior, Related Neurocircuitry, and Metabolism

The majority of studies assessing the metabolic effects of oxytocin administration in humans have used intranasal (IN) oxytocin, which is well tolerated with negligible reported adverse events [211]. Only 1–2% of peripherally synthesized oxytocin penetrates the blood-brain barrier [212] and an even smaller proportion of subcutaneously/intravenously administrated oxytocin reaches the CSF [140]. A recent study has shown that labeled IN oxytocin given to rhesus macaques was later detected in brain regions that lie in the trajectories of the olfactory and trigeminal nerves, suggesting that IN oxytocin delivery into the CNS bypasses the blood-brain barrier [49,213]. Studies in humans [214], non-human primates [215,216], and rodents [217,218] have shown subsequent increase in oxytocin in the CSF 60–75 min [214,216] following IN oxytocin application. Importantly, in nonhuman primates, endogenous oxytocin levels in CSF were not affected following IN or intravenous (IV) labeled oxytocin administration [216], thus providing evidence against a previously proposed feed-forward mechanism of centrally-stimulated oxytocin secretion in response to oxytocin administration [19]. Additionally, labeled exogenous oxytocin given to nonhuman primates intranasally (but not intravenously) was detected in several brain areas that express the OXTR and are involved in the cognitive regulation of appetite in response to visual stimuli of food (e.g., orbitofrontal cortex, amygdala, and striatum) [213]. Pertaining to the potential peripheral metabolic effects of exogenous oxytocin, a concurrent increase in plasma oxytocin concentration has been demonstrated following IN oxytocin administration in humans [219,220] and nonhuman primates [216]; however, the exact mechanism underlying this observation has not yet been clarified.

The hypothesized modulatory role of exogenous oxytocin with respect to appetite regulation has been studied in humans, overall supporting its anorexigenic and metabolically favorable effects. Randomized controlled, double-blind clinical trials have shown that 24 IU of IN oxytocin can either significantly reduce [220,221,222] or have no effect [223,224] on hunger-driven ad libitum caloric consumption tested at a buffet, reflecting homeostatic eating. The acute anorexigenic effect of 24 IU of IN oxytocin in the fasting state has been demonstrated in 25 men across the weight spectrum who were tested 60 min after drug administration [221], in 18 men with obesity evaluated 45 min after administration [222], and in 15 lean men assessed 75 min following oxytocin application [220]. However, it was not demonstrated in 20 young and lean men and 24 healthy-weight men and women tested 45 min after oxytocin administration [223,224]. Whether body weight status and timing of IN oxytocin application interact to affect homeostatic eating remains to be determined. It should also be noted that while objective caloric consumption was reduced by oxytocin in some of these studies, subjective appetite as assessed by the visual analogue scale was unchanged under oxytocin treatment. While studies of oxytocin assessing hunger-driven eating show a reduction or no change in caloric intake, oxytocin consistently reduces hedonic food intake, assessed by palatable snack consumption, in satiated men and women across the weight spectrum [222,223,225,226]. These findings are in line with fMRI studies showing that in men with overweight and obesity, 24 IU of IN oxytocin reduced the blood oxygenation level-dependent (BOLD) signal in response to high-calorie food vs. non-food visual stimuli in the VTA [227], the origin of the mesolimbic dopaminergic reward system and a key hedonic brain region that drives efforts to obtain desired foods [32,228]. Moreover, 24 IU of IN oxytocin attenuated the functional connectivity between the VTA and multiple food motivation brain regions (e.g., anterior insula, oral somatosensory cortex, amygdala, hippocampus) in response to viewing high-calorie foods [229]. Conversely, there was no difference in the functional connectivity between the VTA and these brain areas under oxytocin treatment while subjects were viewing images of low-calorie food and non-food objects, suggesting that oxytocin exerts reward-associated specific inhibitory effects [229]. IN oxytocin has also been shown to suppress hypothalamic fMRI activity in fasting lean participants viewing high- vs. low-calorie food images [224], and in men with excess weight viewing high-calorie vs. non-food visual stimuli [227], suggesting that oxytocin also modulates homeostatic control of appetite regulation. Finally, oxytocin inhibitory effects on food intake could also be explained by enhanced fMRI activation of brain structures known to mediate cognitive control (e.g., the anterior cingulate cortex), as seen in fasting lean individuals while viewing high- vs. low-calorie visual food stimuli [220,230] and in men with excess weight viewing high-calorie vs. non-food visual images [227]. From a behavioral perspective, oxytocin vs. placebo has been shown to increase proactive control in men with overweight and obesity [231]. This effect could potentially contribute to its observed anorexigenic effects in this population, which has been shown to demonstrate impaired cognitive control over impulsive actions [232,233]. There is strong evidence showing that individuals with obesity demonstrate fMRI hyperactivation of reward brain areas triggered by palatable visual food stimuli and it has been proposed that increased activation of the dopaminergic reward circuitry may contribute to overeating behavior and the attainment of excess adiposity [234]. Therefore, oxytocin acute effects on food intake and associated food-processing neurocircuitry could specifically target some of the underlying mechanisms promoting excess adiposity. While one pilot study investigating 8 weeks of oxytocin vs. placebo in humans with obesity showed promising results with significant BMI reduction (3.2 ± 1.9 kg/m^2^) [235], research is currently ongoing to investigate the efficacy and safety of chronic IN oxytocin administration as a weight-loss therapeutic in adults and adolescents (ClinicalTrials.gov Identifiers: NCT03043053 and NCT04551482, respectively).

From a glucose homeostasis standpoint, a single dose of IN oxytocin vs. placebo resulted in attenuated meal-associated glucose excursions in lean men and in non-diabetic individuals with class I obesity (mean BMI 32.10 ± 0.36 kg/m^2^) [222,223,236], but not in a group of non-diabetic men with a wide range of BMIs (18.5–40 kg/m^2^) [221]. While postprandial glucose levels were not blunted in the latter study, oxytocin reduced fasting insulin (but not glucose) levels and therefore the HOMA-IR [221]. Moreover, IN oxytocin administration prior to an oral glucose tolerance test resulted in increased insulin secretion and improved beta cell responsivity in lean men [236], but not in men with class II obesity (BMI 35.3 ± 1.12 kg/m^2^) [237]. In a similar manner, 8 weeks of IN oxytocin did not affect glucose homeostasis parameters in individuals with class II obesity (BMI 36 kg/m^2^) without diabetes despite meaningful weight reduction [235]. Taken together, initial pilot studies suggest that the beneficial glycemic effects of oxytocin administration may decrease with greater adiposity. It should also be noted that to date, human studies examining the effects of oxytocin in obesity have excluded participants with diabetes. Since animal models show that chronic oxytocin treatment can improve diabetes in both diet-induced rodents and in a streptozotocin-induced diabetic mouse model (mimicking T1DM and late-stage T2DM, but without obesity) [235], it will be important to further investigate the therapeutic potential of oxytocin in humans with both obesity and diabetes.

Little is known about the effects of oxytocin on lipid metabolism in humans. An early study conducted in the 1960s showed that IV oxytocin administration in postpartum women induced an increase in nonesterified fatty acid levels concurrent with a decrease in triglyceride concentrations, suggesting increased lipolysis [238]. More recent human studies examined the effects of IN oxytocin. While a single dose of IN oxytocin had no effect on resting energy expenditure measured by indirect calorimetry in both lean individuals [223,236] and those with obesity [221,222,237], it reduced the respiratory quotient and increased fat utilization in men across the weight spectrum [221]. Since oxytocin-induced increase in energy expenditure and BAT thermogenesis has been shown in rodents following chronic administration [83], it is possible that a single dose was not sufficient to demonstrate this effect in humans. Nonesterified fatty acid levels were not affected by a single dose of IN oxytocin given to lean men [236], suggesting once more that a single dose of IN oxytocin given to humans is not sufficient to induce changes in lipid metabolism. In the pilot study mentioned above [235], eight weeks of IN oxytocin administration resulted in reduced LDL and cholesterol levels; however, the confounding beneficial effect of weight loss on lipid metabolism could not be excluded in this study.

The acute effects of oxytocin on levels of other appetite regulating hormones have also been explored, with several studies showing no effect of a single IN oxytocin dose on leptin, ghrelin, and PYY levels [221,222,223] and one study showing increased levels of cholecystokinin (CCK) following oxytocin treatment [221]. The central neurocircuitry in which oxytocin acts involves intricate connections with additional key appetite and energy balance regulating neurons [239]. These include the pro-opiomelanocortin (POMC) [27] and agouti-related protein (AgRP) neurons [240] in the arcuate nucleus of the hypothalamus, NTS neurons that respond to peripheral CCK [241], and GLP-1 cell bodies in the NTS [242]. A recent study has shown that NTS GLP-1 neurons are necessary to mediate the suppressing effects of oxytocin on food intake in mice [243]. It is also known that in rats, intact oxytocin signaling from the PVN to NTS cells that respond to CCK is required for CCK-induced suppression of feeding [241]. Therefore, future human research should closely examine the interaction of oxytocin signaling affecting energy metabolism with additional hormones that modulate eating behavior.

Finally, some of the metabolic effects of chronic oxytocin administration seen in animal models involve modulation of gene transcription and expression. For example, central oxytocin infusion to diet-induced rats increased the expression of enzymes involved in lipolysis and fatty acid beta-oxidation [98] and chronic oxytocin administration in leptin receptor-deficient (db/db) mice led to increased mRNA expression of adipose-tissue inflammatory cytokines as well as anti-inflammatory adipokines [97]. It is possible that some of the metabolic effects of oxytocin that are mediated through induced changes in gene expression occur over many hours and were therefore not detected in human pilot studies looking at the acute effects of a single oxytocin dose. Large-scale human studies assessing chronic oxytocin administration will be instrumental in studying these potential effects.

### 7.2. Oxytocin in Individuals with Obesity Associated with Hypothalamic and Pituitary Impairment

Prader–Willi syndrome (PWS) is a rare neurodevelopmental disorder with an estimated incidence of one in 21,000 newborns [244]. It is characterized by hypotonia, poor feeding, and failure to thrive during the neonatal period and the later development of severe hyperphagia, insatiable eating, and obesity in early childhood [245]. The syndrome is caused by epigenetic and genetic variations resulting in loss of paternally inherent gene expression on the chromosome 15q11.2-q13 [244]. Both human studies and mice models support the importance of this chromosomal area for the development and functionality of both the hypothalamus and pituitary [246]. Brain MRI studies show that the majority of patients with PWS exhibit morphological abnormalities including a small, irregularly shaped pituitary gland as well as modified hypothalamic resting state functional connectivity compared with healthy controls [247,248]. From an endocrine standpoint, PWS can present with multiple pituitary hormonal perturbations, including growth hormone, thyroid, and ACTH deficiencies as well as hypogonadism which can be both central (hypogonadotropic) and peripheral (primary hypogonadism) [246]. PWS is the most common cause of syndromic obesity. Alterations in oxytocin and ghrelin physiology and actions are thought to contribute to the extreme overeating behavior and abnormal body composition characterized by increased adiposity and reduced lean mass [246]. Patients with PWS demonstrate a significant reduction in PVN oxytocin-synthesizing neurons as well as a smaller volume of the PVN itself [132]. Schaaf–Yang syndrome is a rare neurodevelopmental disorder that has a strong clinical overlap with PWS [249]. It is caused by mutations in the *MAGEL2* gene, which is located in the PWS critical region on chromosome 15q11.2-q13 [250] and has a pivotal role in hypothalamic neuroendocrine function [251]. Rodents with *MAGEL2* mutations provide an animal model through which PWS can be investigated. These animals exhibit a reduction in both hypothalamic oxytocin neurons and oxytocin receptors in brain areas involved in learning (e.g., the lateral septum) as well as abnormalities in oxytocin neuronal activity [250,252,253,254]. Moreover, *MAGEL2* knockout mice show additional disrupted arcuate nucleus hypothalamic circuits, including a reduction in POMC-positive cells and α-MSH hormone axons which are critical to induce satiety [255]. Importantly, the PVN and SON oxytocinergic neurons directly project to the POMC neurons in the hypothalamus [27], therefore providing a potential mechanism for oxytocin-mediated disruption in appetite regulation. Similarly to patients with PWS, humans with Schaaf–Yang syndrome demonstrate poor feeding and severe hypotonia in infancy followed by food seeking behavior and obesity in adulthood [256]. Given the strong evidence for central oxytocin neurocircuitry disruption in both *MAGEL2* knockout mice and humans with PWS, oxytocin has been investigated as a therapeutic tool to reduce the hyperphagia and obesity associated with PWS, as well as improve the additional social, behavioral, and cognitive abnormalities that characterize this syndrome [257]. The effects of oxytocin-based therapeutics on eating behavior in patients with PWS have been investigated in three randomized, double-blind, placebo-controlled clinical trials. The first study included 30 children and young adult patients with PWS who received IN oxytocin twice daily for 8 weeks, followed by a wash-out period and an additional 8 weeks of placebo. A third of these patients received either 24 IU (participants older than 16 years of age) or 18 IU (younger individuals) of IN oxytocin twice daily. The other participants in this study received either 40 IU (age > 16 years) or 32 IU (age < 16 years) of the drug twice daily. Oxytocin treatment did not improve reported hyperphagia in this study [258]. In another cross-over study, 25 children with PWS received IN oxytocin twice daily for 4 weeks (24–48 IU per day given over two doses) followed by 4 weeks of placebo. A significant improvement in reported eating behavior was found in the young children, but not in older patients; however, BMIs were unaffected by the treatment [259]. In the third study, IN carbetocin, a longer-acting oxytocin analogue given three times daily for 2 weeks, improved hyperphagia in youth (10–18 years of age) with PWS compared with placebo [260]. Body mass was not assessed in this study. Taken together, these studies suggest that early rather than late intervention with oxytocin-based therapeutics may be beneficial to improve eating behaviors in patients with PWS, as indicated by caregiver’s reports and standardized questionnaires. A phase 3 study examining the effects of IN carbetocin is currently ongoing (NCT03649477).

Oxytocin treatment has also been investigated as a potential therapeutic agent for hypothalamic obesity. This condition most commonly occurs in the setting of brain tumors, specifically craniopharyngioma, requiring surgical management [261]. The neuroanatomical location of craniopharyngioma tumors, which develop from remnant epithelial cells of Rathke’s pouch, frequently affects the hypothalamic and/or pituitary regions, therefore critically affecting energy balance regulation [262]. Patients with hypothalamic obesity demonstrate extreme hyperphagia, decreased energy expenditure, and rapid weight gain, which are rarely controlled with lifestyle modifications [263]. Craniopharyngioma is associated with lower baseline salivary oxytocin levels in patients with vs. without hypothalamic damage and compared with healthy controls [264]. Additionally, patients with craniopharyngioma demonstrate attenuated salivary oxytocin increase in response to exercise compared with healthy individuals [264]. Moreover, individuals with craniopharyngioma exhibit inverse relationships between BMI and change in salivary oxytocin levels pre- vs. post-meal which are not found in healthy controls, thus further supporting altered oxytocin signaling in these patients, impacting eating behavior and weight [265]. The smallest change in oxytocin levels in response to both exercise and meal stimuli was seen in the patients with the highest BMI, thus providing a potential link between perturbated oxytocin pathways and obesity related to craniopharyngioma [264,265]. In a case report of a 13-year-old male with hypothalamic obesity secondary to craniopharyngioma resection, IN oxytocin given for 10 weeks resulted in a significant improvement in hyperphagia and meaningful weight reduction [266]. An additional 38 weeks of oxytocin treatment combined with IN naloxone resulted in further weight reduction concurrently with decreased need for the patient’s care givers to control his eating [266]. Since oxytocin receptors are also located in key energy balance brain regions outside of the pituitary-hypothalamic area, IN oxytocin could potentially induce beneficial metabolic effects in this population, compensating for the hypothalamic insult. A randomized, placebo-controlled study investigating the effects of prolonged IN oxytocin administration as a weight-loss therapy for youth with hypothalamic obesity is currently underway (NCT02849743).

Alterations in oxytocin status have also been demonstrated in men with hypopituitarism due to brain tumors or surgery [267,268]. Fasting plasma oxytocin levels were significantly lower in men with hypopituitarism associated with CDI compared with men with hypopituitarism who had preserved vasopressin secretion and compared with healthy controls [267]. In participants with CDI, a trend towards an association between higher BMI and lower oxytocin levels was found, suggesting the possible involvement of oxytocin metabolism in the weight status of these patients. Similarly, adults with hypopituitarism with and without CDI were found to have lower salivary oxytocin levels compared with healthy controls [268]. Whether oxytocin perturbations in patients with hypopituitarism underly additional metabolic disruptions which could be treated with oxytocin-replacement therapy remains to be investigated.

## 8. Oxytocin as a Therapeutic Agent in Obesity and Metabolic Syndrome—Promise, Fundamental Challenges and Future Directions

There is increasing evidence that oxytocin participates in the regulation of eating behavior, energy balance, and metabolism. Given the growing global obesity epidemic and projected prevalence of obesity, predicting that nearly 1 in 4 adults in the United States will have severe obesity by 2030 [269], oxytocin has emerged as a potential therapeutic agent to treat obesity. Animal studies strongly support the weight-reducing properties of oxytocin, via suppression of food intake, particularly reward-driven eating, and increase in thermogenesis and energy expenditure [9]. The OXTR has an important role in the musculoskeletal system. It is needed for muscle maintenance and regeneration, to promote bone mineralization and to minimize adipose tissue generation from mesenchymal stem cells, thus overall exerting beneficial effects on body composition. These findings together with a reassuring safety and tolerability profile [211] catalyzed the conception of multiple human research studies designed to assess the metabolic and neurophysiological effects of oxytocin. Many of these trials provided proof-of-concept investigations supporting the potential of exogenous oxytocin to reduce food intake and improve metabolic parameters. Moreover, oxytocin has been shown to suppress the fMRI response to palatable visual food stimuli in the hypothalamus, which has a role in homeostatic regulation of food intake, as well as additional brain areas representing reward processing (e.g., VTA, orbitofrontal cortex, insula, hippocampus, and amygdala). Finally, oxytocin administration resulted in the enhancement of fMRI activation of cognitive-control brain areas (e.g., anterior cingulate gyrus) in response to palatable food images and improved cognitive control in a behavioral task, thus potentially supporting the role of oxytocin in inhibition of food intake [221,223,237,270]. Still, important challenges remain in translating this body of work to routine patient care. A comprehensive understanding of endogenous oxytocin physiology is yet to be attained, with the compilation and synthesis of previous studies assessing oxytocin concentrations in body fluids being limited by the lack of a reliable, validated, and reproducible measurement technique [135]. Whether oxytocin dynamics are altered in obesity and diabetes is currently not well understood and should be clarified to enhance the development of oxytocin-based treatment for these conditions. The peripheral vs. central actions of exogenous IN oxytocin also deserve additional investigation to improve our understanding of mechanisms underlying oxytocin effects on metabolic regulation. A precise and delineated description of endogenous oxytocin involvement in human insulin secretion, lipid metabolism, thermogenesis, and musculoskeletal function is crucially needed to form an in-depth understanding of the potential effects of oxytocin treatment on obesity-associated co-morbidities. It is currently not well understood whether oxytocin secretion follows a circadian rhythm in humans which could potentially affect usage of synthetic oxytocin for therapeutic purposes. Non-human primates demonstrate diurnal rhythmic variations in CSF oxytocin levels, which are probably not dependent on the integrity of the suprachiasmatic nuclei (SCN) [271]; however, in rats, a neural pathway from SCN AVP neurons to PVN oxytocin neurons exists and it may explain light-induced food-intake suppression [272]. A circadian rhythm of CSF oxytocin levels has not been demonstrated in patients admitted to the intensive care unit who had an external ventricular shunt [137], but data regarding central oxytocin diurnal variation in healthy subjects and the implication concerning the usage of exogenous oxytocin are lacking. Given the fact that long-term oxytocin treatment in rodents was associated with sustained weight reduction and improved body composition despite weakening of the anorexigenic effect and most probably due to increased energy expenditure [118], it will be important to fully elucidate the involvement of oxytocin in energy balance in humans. It should be noted that the vast majority of animal and human studies exploring the role of oxytocin in appetite regulation have been conducted in males only. There is evidence showing that central appetite regulation is sexually dimorphic [273,274], and therefore targeted investigations of oxytocin effects in females would be critical to pursue in order to allow the development of this drug as an effective anti-obesity pharmacotherapy. To date, most clinical trials used an IN formulation of synthetic oxytocin which was shown to increase both CSF and plasma oxytocin levels. However, the pharmacokinetics of this formulation, the time to reach peak levels, and the possibility of tachyphylaxis are yet to be clearly defined. Long-acting oxytocin receptor agonists are currently being investigated [275,276]. Finally, potential cross-reactivity between oxytocin and vasopressin receptors is possible due to the homology between the two neuropeptides as well as their receptors [277,278]. In addition to possible cross-reactivity-related side effects concerning sodium–water balance and blood pressure regulation (e.g., hyponatremia and hypertension), there is evidence suggesting that oxytocin and vasopressin exert opposing effects on emotional behavior [279]. More specifically, while oxytocin induces anxiolysis and anti-depressive effects, vasopressin promotes anxiety and depression [279]. Future studies should monitor for these potential side effects and form a clear understanding of the relation between exogenous oxytocin dosing and cross-reactivity with the vasopressin receptor. Oxytocin signaling is exceptionally conserved over species and evolution, supporting its pivotal role in organism function. Ongoing large-scale, well-designed clinical trials will significantly contribute to our understanding of the potential for oxytocin-based therapies to improve overeating behavior, excess adiposity, and associated sequela.
